# Effects of Plant-Based Supplement on Oxidative Stress of Honey Bees (*Apis mellifera*) Infected with *Nosema ceranae*

**DOI:** 10.3390/ani13223543

**Published:** 2023-11-16

**Authors:** Nemanja M. Jovanovic, Uros Glavinic, Marko Ristanic, Branislav Vejnovic, Tamara Ilic, Jevrosima Stevanovic, Zoran Stanimirovic

**Affiliations:** 1 Department of Parasitology, Faculty of Veterinary Medicine, University of Belgrade, Bul. Oslobodjenja 18, 11000 Belgrade, Serbia; nmjovanovic@vet.bg.ac.rs (N.M.J.); tamara@vet.bg.ac.rs (T.I.); 2 Department of Biology, Faculty of Veterinary Medicine, University of Belgrade, Bul. Oslobodjenja 18, 11000 Belgrade, Serbia; uglavinic@vet.bg.ac.rs (U.G.); mristanic@vet.bg.ac.rs (M.R.); zoran@vet.bg.ac.rs (Z.S.); 3 Department of Economics and Statistics, Faculty of Veterinary Medicine, University of Belgrade, Bul. Oslobodjenja 18, 11000 Belgrade, Serbia; branislavv@vet.bg.ac.rs

**Keywords:** dietary supplement, *Nosema ceranae*, oxidative stress, gene expression, vitellogenin

## Abstract

**Simple Summary:**

Microsporidia *Nosema ceranae* is an obligate intracellular parasite of the honey bee and causes nosemosis, a disease with negative effects on the health, reproductive and productive capabilities of bee colonies. The aim of this study was to investigate the effects of a plant-based supplement branded as “B+” on honeybees in a laboratory experiment. Food supplement branded as “B+” showed the potential to positively influence bee survival. Supplemental feeding reduced *N. ceranae* infection level. Treatment significantly decreased oxidative stress in *Nosema*-infected bees. Vitellogenin gene expression was the highest in the supplement-fed group.

**Abstract:**

One of the most important approaches in the prevention and treatment of nosemosis is the use of herbal preparations as food supplements for bees. Therefore, the aim of this study was to investigate the effects of a plant-based supplement branded as “B+” on honeybees in a laboratory experiment. Four experimental groups were established: treated group (T), *N. ceranae*-infected and treated group (IT), *N. ceranae*-infected group (I) and non-infected group (NI). Survival, *N. ceranae* spore load and oxidative stress parameters together with expression levels of antioxidant enzyme genes and vitellogenin gene were monitored. The mortality in the T, IT and NI groups was significantly (*p* < 0.001) lower than in than in the I group. Within *Nosema*-infected groups, the IT group had a significantly lower (*p* < 0.001) number of *N. ceranae* spores than the I group. In addition, expression levels of genes for antioxidant enzymes were lower (*p* < 0.001) in the IT group compared to the I group. The concentration of malondialdehyde and the activities of antioxidant enzymes (superoxide dismutase, catalase and glutathione S-transferase) were significantly lower (*p* < 0.001) in the IT group compared to the I group. No negative effects of the tested supplement were observed. All these findings indicate that the tested supplement exerted beneficial effects manifested in better bee survival, reduced *N. ceranae* spore number and reduced oxidative stress of bees (lower expression of genes for antioxidant enzymes and oxidative stress parameters).

## 1. Introduction

The Western honey bee (*Apis mellifera*) is the most significant pollinator worldwide [1]. Appropriate pollination increases the quality and quantity of fruit, nut, vegetable, seed, oil and fiber crops [1,2]. On the other hand, the nutrition of honey bees depends on the collected nectar and pollen, which are then transformed into honey and bee bread in the hive, providing carbohydrates, proteins, lipids, vitamins and minerals [3]. Food availability and nutritional balance play a central role in regulating physiological processes including resilience to oxidative stress, adequate immune response, interaction with pathogens, brood production and development and winter survival of colonies [4].

Various abiotic and biotic stressors can negatively affect bees, and several of them have synergistic effects on the health of bee colonies and can reduce their survival [4,5]. Numerous factors that have been contributing to bee colony losses are described, most notably the diseases of infectious and parasitic etiology, in-hive chemical substances, agrochemicals, beekeeping management, climate change, modified land use and changes in land cultivation [4,6,7,8,9]. The lack of natural plant diversity in agricultural systems can limit the availability of pollen and nectar, which has a significant impact on pollinators. Consequently, nutritional stress has a direct impact on the behavior and physiology of bees, affects honey bee lifespan, their immunocompetence and resistance to pathogens [4,10]. This type of stress typically affects honey bee colonies over the long term, which is detrimental to bee health and their productive and reproductive characteristics [5,11,12]. Poor nutrition can impair bees’ resistance to other stressors and even increase their mortality [13,14]. Infection with the parasitic microsporidium *Nosema ceranae,* recently redefined to *Vairimorpha* [15], is one of the most frequent biotic stressors of honey bees worldwide [16,17,18,19,20], including Serbia [21]. It has been shown that *N. ceranae* suppresses the honey bee immunity and causes oxidative stress [4,22,23,24,25], and energetic stress [26,27], and decreases honey bee colony strength [5] and their hygienic behavior [4]. Many studies indicate that virulence of *N. ceranae* increases in combination with other stressors, including chemical substances and other pathogens/parasites [28,29,30,31]. Such interaction between stressors could explain the widespread colony losses reported in different parts of the world.

Low-quality bee forage and/or a nutritional deficiency promote the multiplication of *N. ceranae* [4,27,32,33]. Good Beekeeping Practice, especially the hygienic aspect, is crucial for the prevention of endoparasite expansion in hives [34]. In addition, supplementing bee diet with natural-based preparations based on botanical essential oils, organic acids, mushroom polysaccharides, bacteria and their metabolites are also very important strategies in the prevention and treatment of nosemosis [4,5,22,23,24,25,35,36,37]. Taking everything into consideration, the aim of this research was to examine the effects of a plant-based supplement of the brand name “B+” added to the bee diet in a laboratory experiment by analyzing (i) bee survival, (ii) the dynamics of *N. ceranae* development, (iii) the expression levels of antioxidant enzyme-encoding genes and vitellogenin gene and (iv) oxidative stress parameters in bees infected with microsporidia *N. ceranae*.

## 2. Material and Methods

### 2.1. Supplement

In this experiment, the herbal supplement, with the brand name “B+” (Certificate No. RS 30242), containing wheat bran, essential oils, cinnamon, dextrose, brewer’s yeast, lecithin, saturated and unsaturated fatty acids, plant proteins, essential amino acids, lipids and B vitamin mineral complex was tested [5]. The feeding solution was made of 1 g of supplement per liter of sugar (saccharose) syrup 50% (*w*/*v*) as recommended by the manufacturer’s instructions.

### 2.2. Experimental Design

The bees used in this experiment originated from the experimental apiary of the Faculty of Veterinary Medicine, University of Belgrade. Bee colonies were clinically healthy, with no signs of disease of adult bees or brood.

Frames with sealed brood were taken from five randomly chosen hives, transported to the laboratory and placed into the incubator at 34 ± 1 °C and 66 ± 1% relative humidity as described in Glavinic et al. [22]. After twenty-four hours, newly emerged bees were placed into the specially designed cages described by Glavinic et al. [22]. Each cage contained 110 bees. Bees were fed ad libitum with sugar (saccharose) syrup 50% (*w*/*v*).

The following experimental groups were formed: treated group—bees fed with the addition of test supplement (T); treated and infected group—bees fed with the addition of test supplement and infected with *N. ceranae* spores (IT). In addition, two control groups were established: *N. ceranae*-infected group (I), and non-infected group, containing non-infected bees (NI). Each group included six cages and the experiment was repeated twice.

On the third day of the experiment, workers in groups IT and I were infected with *N. ceranae* spores, whose species affiliation had been previously confirmed using the PCR technique as described in Stevanovic et al. [21]. Inoculum preparation (concentration 10^6^ spores/mL) and infection of bees were conducted as described in Glavinic et al. [22].

From each cage, on days 7 and 14, 10 bees were sampled for RNA extraction and measurements of transcript levels of genes for antioxidant enzymes and *vitellogenin*, 15 bees for measurement of oxidative stress parameters and 15 bees for *N. ceranae* spore count. The remaining 30 bees in each cage were used for survival monitoring until the end of the experiment. Each day, dead bees were removed and their quantity in each cage recorded in order to estimate the survival rate.

### 2.3. Nosema Spore Counting

The abdomens of the bees were individually macerated in 1.5 mL tubes with 3 mm tungsten carbide beads (Qiagen, Valencia, Germany) in a TissueLyser II (Qiagen, Valencia, Germany) for 1 min at 25 Hz [25]. *Nosema ceranae* spores were quantified using a haemocytometer according to Cantwell [38].

### 2.4. Gene Expression Analyses

The extraction of total RNA from the sampled bees was performed using the Quick-RNA MiniPrep Kit (Zymo Research, Irvine, CA, USA) according to the manufacturer’s instructions. For cDNA synthesis, 1000 ng of RNA per sample were reverse-transcribed using RevertAid™First Strand cDNA Synthesis Kit (Thermo Fisher Scientific, Vilnius, Lithuania).

Real-Time PCR analysis of gene expression levels was performed using the commercial kit KAPA SYBR^®^ FAST Master Mix (2X) Universal” (KAPA Biosystems, Boston, MA, USA). Gene expression levels for antioxidant enzymes directly involved in the scavenging of superoxide radicals and H_2_O_2_ were investigated. These enzymes included *cytoplasmic Cu-Zn superoxide dismutase* (Cu/Zn SOD), *mitochondrial Mn superoxide dismutase* (MnSOD), *catalase* (CAT) and *glutathione S-transferase* (GST). In addition, transcript levels of *vitellogenin* gene were also monitored. The primers for target genes and an internal control gene are listed in Table 1. Quantification was performed via Rotor-Gene Q 5plex (Qiagen, Valencia, CA, USA) for 45 cycles at 95 °C (5 s), 60 °C (30 s) and 72 °C (5 s) after initial denaturion at 95 °C for 2 min. Fluorescence was measured at the end of the annealing stage of every cycle. The 2^−ΔΔCt^ method as described in Glavinic et al. [22] was used. The median value of the NI group served as a calibrator, while *β*-*actin* was used as an internal control gene [22].

### 2.5. Analyses of Oxidative Stress Parameters

The spectrophotometric analyses described in Dubovskiy et al. [41] were used for measurements of the following oxidative stress parameters: activities of the antioxidative enzymes superoxide dismutase (SOD), catalase (CAT) and glutathione S-transferase (GST), and the concentrations of malondialdehyde (MDA). On each sampling day (7th and 14th), pools of 15 bees collected from each cage were used for analyses on a UV/VIS Spectrophotometer BK-36 S390 (Biobase Biodustry, Shanghai, China).

### 2.6. Statistic Analyses

The assessment of bee survival was conducted by monitoring the daily count of dead bees within each experimental group. The data of survival distribution were obtained via the Kaplan–Meier survival estimator and compared using the log-rank test.

The results of *N. ceranae* spore loads, expression levels of genes (for GST, MnSOD, CuZnSOD, CAT and *vitellogenin*) and values of oxidative stress parameters (GST, SOD and CAT activities and MDA concentration) were tested for normality via Shapiro–Wilk’s test.

The results of *N. ceranae* spore loads were compared between groups using the unpaired *t*-test, and within each group using paired *t*-test.

Given that some data for gene expression levels (MnSOD, CuZnSOD, CAT and *vitellogenin*) were not normally distributed (Shapiro–Wilk’s test, *p* < 0.05), adequate transformations were made: MnSOD and CuZnSOD were expressed as log10(y + 5), CAT was expressed as log10(y + 3) and for the *vitellogenin*, log10(y + 10) transformation was applied. Gene expression levels and values of oxidative stress parameters were compared via two-way ANOVA with repeated measures in one factor, followed by Sidak’s test within and Tukey’s test between groups over time. Statistical significance levels were calculated: *p* < 0.05, *p* < 0.01 and *p* < 0.001. Statistical analysis was carried out using statistical software GraphPad Prism version 7 (GraphPad, San Diego, CA, USA).

## 3. Results

### 3.1. Bee Survival

**Figure 1:**
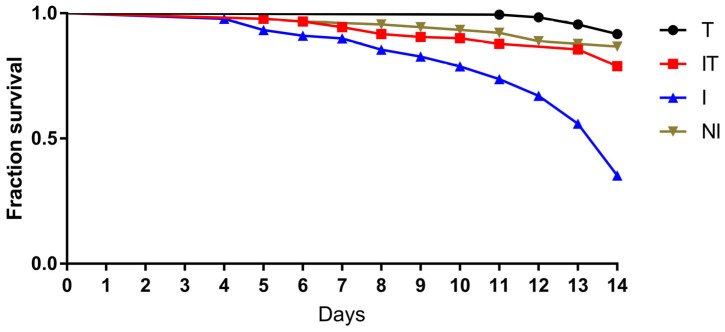
Survival of bees in treated group (T), *N. ceranae*-infected and treated group (IT), *N. ceranae*-infected group (I) and non-infected group (NI).

The number of dead bees at the end of the experiment was significantly higher (*p* < 0.001) in the infected group (I) compared to treated (T), treated and infected (IT) and non-infected (NI). A significantly higher (*p* < 0.001) number of dead bees was also recorded in the IT group compared to the T group (Figure 1, Supplementary Table S1).

### 3.2. Quantification of N. ceranae Spore Loads

**Figure 2:**
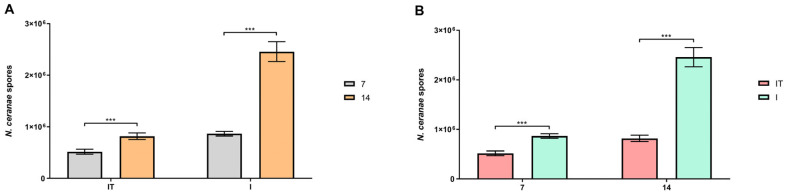
Number of *N. ceranae* spores in infected group (I) and infected and treated group (IT). (**A**) Comparison of *N. ceranae* spore loads within groups between samples collected on days 7 and 14. (**B**) Comparison of *N. ceranae* spore loads between groups on samples collected on days 7 and 14. *** *p* < 0.001.

On both sampling occasions, days 7 and 14, neither the group fed with the test supplement nor the non-infected group contained *N. ceranae* spores. Based on a paired *t*-test, the number of spores in groups IT and I on day 14 was significantly higher (*p* < 0.001) than on day 7 (Figure 2A). Using an unpaired *t*-test to compare the results between groups, a significantly lower (*p* < 0.001) number of spores was detected in the IT group compared to the I group on both days 7 and 14 (Figure 2B).

### 3.3. Gene Expression Analyses

**Figure 3:**
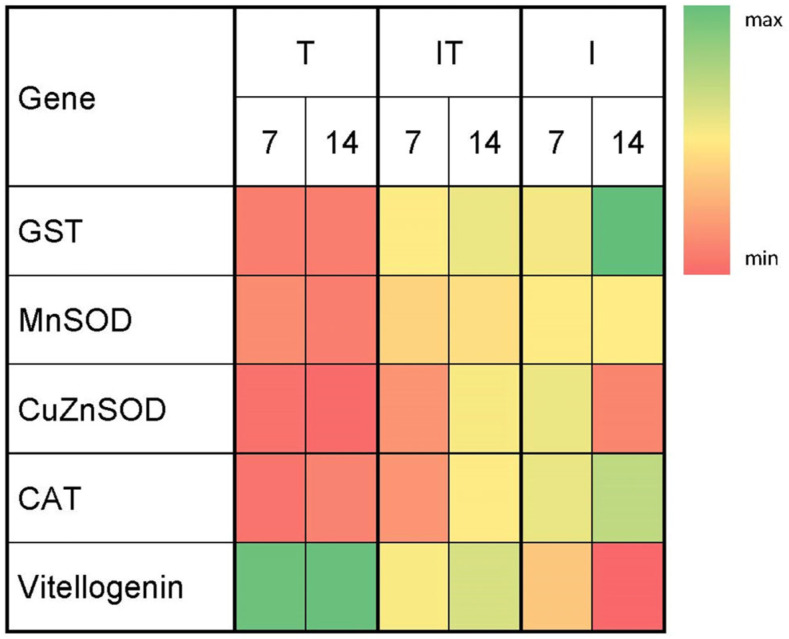
Heat map of median values of relative genes’ expression levels, *cytoplasmic Cu-Zn superoxide dismutase* (Cu/ZnSOD), *mitochondrial Mn superoxide dismutase* (MnSOD), *catalase* (CAT), *glutathione S-transferase* (GST) and *vitellogenin* at different time points (at days 7 and 14) in the experimental groups. Treated group (T), *N. ceranae*-infected and treated group (IT), *N. ceranae*-infected group (I) and non-infected group (NI).

In the infected group, according to Sidak’s test, the level of GST gene expression was significantly higher (*p* < 0.001) on day 14 compared to day 7 (Figure 3, Supplementary Figure S1A). On day 14, according to Tukey’s test, the value of GST gene expression was significantly higher (*p* < 0.001) in the I group than in the IT and T groups (Figure 3, Supplementary Figure S1B). Gene expression levels of MnSOD were not significant between sampling occasions (on days 7 and 14) within each group and between groups at each sampling occasion (Supplementary Figure S3A,B). On day 7, the highest level of Cu-Zn SOD gene expression was found in the I group, which was significantly higher (*p* < 0.01 and *p* < 0.05) compared to the T and IT groups, respectively (Figure 3, Supplementary Figure S2B). The level of CAT gene expression on both sampling days (7 and 14) was significantly higher (*p* < 0.01 and *p* < 0.001) in group I when compared to the IT and T groups, respectively (Figure 3, Supplementary Figure S4B). On days 7 and 14, the expression level of the *vitellogenin* gene was significantly higher in group T (*p* < 0.05, *p* < 0.01 and *p* < 0.001) than in groups IT and I, respectively (Figure 3, Supplementary Figure S5B).

### 3.4. Oxidative Stress Parameters

**Figure 4:**
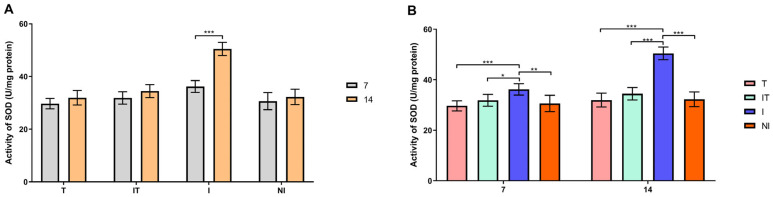
Superoxide dismutase (SOD) activity: (**A**) comparisons between sampling occasions (on days 7 and 14) within each group and (**B**) comparisons between groups at each sampling occasion. * *p* < 0.05; ** *p* < 0.01; *** *p* < 0.001; treated group (T), *N. ceranae*-infected and treated group (IT), *N. ceranae*-infected group (I) and non-infected group (NI).

**Figure 5:**
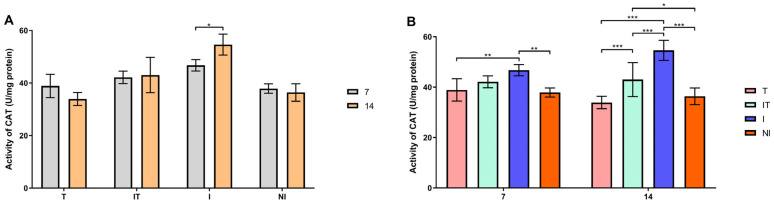
Catalase (CAT) activity: (**A**) comparisons between sampling occasions (on days 7 and 14) within each group and (**B**) comparisons between groups at each sampling occasion. * *p* < 0.05; ** *p* < 0.01; *** *p* < 0.001; treated group (T), *N. ceranae*-infected and treated group (IT), *N. ceranae*-infected group (I) and non-infected group (NI).

**Figure 6:**
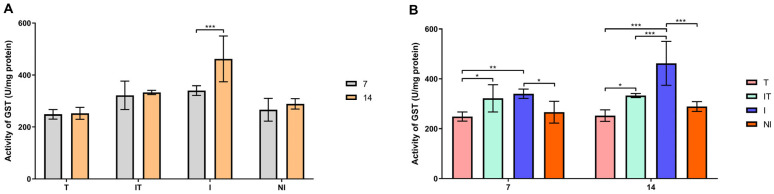
Glutathione-S transferase (GST) activity: (**A**) comparisons between sampling occasions (on days 7 and 14) within each group and (**B**) comparisons between groups at each sampling occasion. * *p* < 0.05; ** *p* < 0.01; *** *p* < 0.001; treated group (T), *N. ceranae*-infected and treated group (IT), *N. ceranae*-infected group (I) and non-infected group (NI).

**Figure 7:**
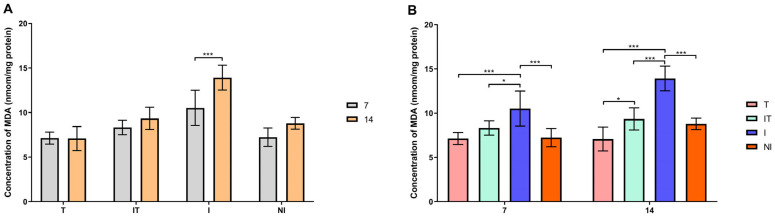
Concentration of malondialdehyde (MDA): (**A**) comparisons between sampling occasions (on days 7 and 14) within each group and (**B**) comparisons between groups at each sampling occasion. * *p* < 0.05; *** *p* < 0.001; treated group (T), *N. ceranae*-infected and treated group (IT), *N. ceranae*-infected group (I) and non-infected group (NI).

According to Sidak’s test, the activities of GST, SOD and CAT and the concentration of malondialdehyde (MDA) were significantly higher (*p* < 0.05 and *p* < 0.001) in the infected group on day 14 compared to day 7 (Figure 4A, Figure 5A, Figure 6A and Figure 7A). On day 7, Tukey’s test revealed that the activities of the examined enzymes GST, CAT and SOD were significantly higher (*p* < 0.05 and *p* < 0.001) in the I group compared to the T, IT and NI groups. On day 14, however, the most significant changes in enzyme activity were determined. GST, CAT and SOD enzyme activities were significantly higher (*p* < 0.001) in group I compared to groups T, IT and NI. Also, Tukey’s test revealed that GST activity was higher (*p* < 0.05) in the IT group than in the T group, whereas CAT activity was higher (*p* < 0.05 and *p* < 0.001) in the IT group than in the T and NI groups (Figure 4B, Figure 5B and Figure 6B). On days 7 and 14, the concentration of malondialdehyde was significantly higher (*p* < 0.001) in the I group than in the T, IT and NI groups. At the end of the experiment, the concentration of MDA in the IT group was significantly higher (*p* < 0.05 and *p* < 0.001) than in the T group (Figure 7B).

## 4. Discussion

Several investigations over the past ten years have shown positive effects of various supplements and organic extracts on the health and survival of *N. ceranae*-infected bees [22,24]. In a review paper by Marín-García et al. [37], numerous plant extracts with a positive impact on the survival of *Nosema*-infected bees were described. In our study, a higher rate of survival was detected in the group of bees that were simultaneously infected with *N. ceranae* and received the supplement (IT) compared to the infected group (I) where *N. ceranae* infection caused the highest bee mortality. The absence of significant differences in bee mortality between the non-infected group (NI) and bees fed with tested supplement (T) confirms that the supplement “B+” has a positive effect on bee survival.

The presence of *N. ceranae* spores in the infected groups (I and IT) and their absence in the non-infected groups (T and NI) show that there was no cross-contamination between the experimental groups. On days 7 and 14, the number of *N. ceranae* spores was significantly higher in the infected group (I) than in the infected and supplemented group (IT). This could be explained by the fact that certain substances in supplements (vitamins and minerals) tested by other authors [22,24], which have similar ingredients to the “B+” supplement, exerted an immunostimulatory effect which probably maintains a lower infection level compared to the infected group.

In the IT group of this study, an increase in *N. ceranae* spore number on day 14 compared to day 7 was found. In several studies [42,43,44], it has been shown that feeding bees with pollen/bread simultaneously increases the development of the endoparasite *N. ceranae* but also increases the survival rate of bees relative to those fed only with syrup without a protein source. This indicates that bees that are adequately fed, i.e., not under nutritional stress, can effectively combat bee pathogens which is in accordance with the results of this research because survival was better in the IT group compared to the I group. In accordance with that, our previous results [5] revealed that colonies fed with the supplement “B+” had a decreased level of *N. ceranae* infection. Additionally, in these colonies, there were more worker bees, greater areas of open and sealed brood, and more food reserves (honey and pollen/bread).

Analyzing the results of the expression levels of genes important for the synthesis of antioxidant enzymes at two sampling occasions (on day 7 and day 14) revealed significant differences between the experimental groups. Several studies have demonstrated that the production of reactive oxidative species (ROS) is one of the most important parts of immune response to the presence of pathogens, including microsporidum *N. ceranae* [22,24,45]. Reactive oxidative species, which are effective antimicrobial compounds, are generated in the oxidation-reduction processes. An increase in the oxidation-reduction process in the gastrointestinal epithelial cells of bees infected with *N. ceranae* indicates an increase in the production of ROS in response to infection [22,24,45]. In our experiment, a higher level of gene expression for CuZnSOD and CAT was detected on day 7 in the infected group (I) compared to the other two groups (T and IT), as a result of the bees’ response to *N. ceranae* infection. At the end of the experiment on day 14, an increase in the activity of genes for GST and CAT confirmed that this microsporidium causes overexpression of oxidation-related genes [45]. In our previous study [46], in which the effect of lithium citrate was tested, the expression levels of genes for antioxidant enzymes depended on pathogen load. In contrast to the infected group (I), bees that were infected and supplemented (group IT) had lower gene transcript levels. The reason for this is undoubtedly the lower number of *N. ceranae* spores due to supplementation, but we cannot overlook the fact that certain substances of the tested supplement “B+” act as antioxidants and neutralize ROS. Expression levels of genes for antioxidant enzymes in group T, which were not different compared to group IT, provide additional confirmation of the tested supplement’s positive effects.

By analyzing the activities of antioxidant enzymes (GST, SOD and CAT) and the MDA concentration, it was determined that their values were highest in the I group. Our findings which demonstrate that *Nosema* infection induces oxidative stress in honeybees are in accordance with earlier research [22,23,24,25]. Dussaubat et al. [45] stated that the generation of ROS with antimicrobial properties by bees is ineffective against *N. ceranae* infection, which was determined in our experiment by counting *Nosema* spores in group I. In contrast, the activities of the observed parameters were lower in the IT group compared to the I group. Consequently, it can be concluded that nutrition with the addition of our investigated supplement (“B+”) likely plays an important role in the antioxidant protection of bees, which is consistent with the findings of Glavinic [23] that bees infected with *N. ceranae* and fed with dietary amino acid and vitamin complex (which, among other things, also contains B vitamin complex) have a lower level of oxidative stress.

The tested supplement (“B+”) also had a positive effect on the expression of the gene for *vitellogenin*, which was highest in group T, indicating that the expression of this gene is related to the nutritional quality of the bees’ diet. Given that this gene is expressed in the fat body, which is the primary nutrient storage [47], it is reasonable to expect a rise in *vitellogenin* expression due to nutritive factors. Pollen feeding increases the transcription of genes involved in the expression of *vitellogenin* [33,48]. For example, the diet with lipid rich pollen (Erica sp. pollen) had the greatest influence on the expression of this gene [47]. In groups infected with *N. ceranae* microsporidium in our study (IT and I), although no difference was found between the groups, a higher level of *vitellogenin* gene expression was observed in the IT group, similar to how pollen feeding increased the transcription of genes associated with *vitellogenin* expression in the studies of Borba et al. [48] and Castelli et al. [33]. In contrast, supplemental feeding of queens with FeedBee^®^ did not influence *vitellogenin* gene expression [49]. It was proved that *Nosema* infection suppressed *vitellogenin* gene expression [22,23,24,25]. In accordance with the findings of Dussaubat et al. [45] and Glavinic et al. [22,24,25], lower levels of *vitellogenin* gene expression observed in this study are attributable to the pathogenic effect of microsporidium *N. ceranae*, reflected in increased oxidative stress and decreased *vitellogenin* gene expression levels.

## 5. Conclusions

Our findings indicate that the tested supplement investigated in a laboratory (cage) experiment showed the potential to (i) positively influence bee survival; (ii) reduce *N. ceranae* infection level; (iii) reduce oxidative stress of infected bees by decreasing the activity of antioxidative enzymes; and (iv) simultaneously reduce the expression of the genes that encode them.

## Figures and Tables

**Table 1:** Primers used for real-time polymerase chain reaction.

Primer	Sequence 5′–3′	Reference
Cu/ZnSOD-F	TCAACTTCAAGGACCACATAGTG	[39]
Cu/ZnSOD-R	ATAACACCACAAGCAAGACGAG	
MnSOD-F	GTCGCCAAAGGTGATGTCAATAC	[39]
MnSOD-R	CGTCTGGTTTACCGCCATTTG	
GST-F	AGGAGAGGTGTGGAGAGATAGTG	[39]
GST-R	CGCAAATGGTCGTGTGGATG	
CAT-F	TTCTACTGTGGGTGGCGAAAG	[39]
CAT-R	GTGTGTTGTTACCGACCAAATCC	
VgMC-F	AGTTCCGACCGACGACGA	[40]
VgMC-R	TTCCCTCCCACGGAGTCC	
β-actin-F	TTGTATGCCAACACTGTCCTTT	[40]
β-actin-R	TGGCGCGATGATCTTAATTT	

F, forward; R, reverse; GST, *glutathione S-transferase*; Cu/ZnSOD, *cytoplasmic Cu-Zn superoxide dismutase*; MnSOD, *mitochondrial Mn superoxide dismutase*; CAT, *catalase*; VgMC, *vitellogenin*; *β*-*actin*, beta actin.

## Data Availability

Data are contained within the article.

## Supplementary Materials

The following supporting information can be downloaded at: https://www.mdpi.com/article/10.3390/ani13223543/s1, Table S1: Survival of bees in treated group (T), *N. ceranae*-infected and treated group (IT), *N. ceranae*-infected group (I) and non-infected group (NI). Figure S1: Mean values for the relative gene expression of *glutathione S-transferase*; Figure S2: Mean values for the relative gene expression of *cytoplasmatic Cu-Zn superoxide dismutase*. Figure S3: Mean values for the relative gene expression of *mitochondrial Mn superoxide dismutase*; Figure S4: Mean values for the relative gene expression of *catalase*; Figure S5: Mean values for the relative gene expression of *vitellogenin*.
