# Supplementary material for: Effects of Plant-Based Supplement on Oxidative Stress of Honey Bees (Apis mellifera) Infected with Nosema ceranae
Source: Animals (Basel). 2023 Nov 16;13(22):3543. doi: 10.3390/ani13223543 (PMC10668651; doi:10.3390/ani13223543)
Supplement: Supplementary file 1 [file animals-13-03543-s001.zip › animals-2684543-supplementary.pdf]

Supplementary Table S1. Survival of bees in treated group (T), *N. ceranae*-infected and treated group (IT), *N. ceranae*-infected group (I) and non-infected group (NI).

Group	Treated (T)	Infected and treated (IT)	Infected (I)	Non-infected (NI)
Treated (T)		p<0.001 ***	p<0.001 ***	0.0964
Infected and treated (IT)			p<0.001 ***	0.0592
Infected (I)				<0.001 ***
Non-infected (NI)				

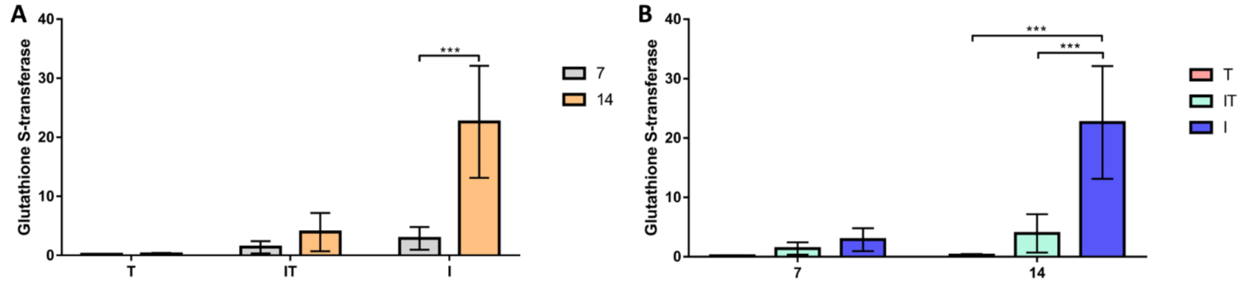

Supplementary Figure. S1. Mean values for the relative gene expression of glutathione S-transferase: (A) comparisons between sampling occasions (on day 7 and 14) within each group and (B) comparisons between groups at each sampling occasion. ***p < 0.001; Treated group (T), *N. ceranae*-infected and treated group (IT), *N. ceranae*-infected group (I) and non-infected group (NI).

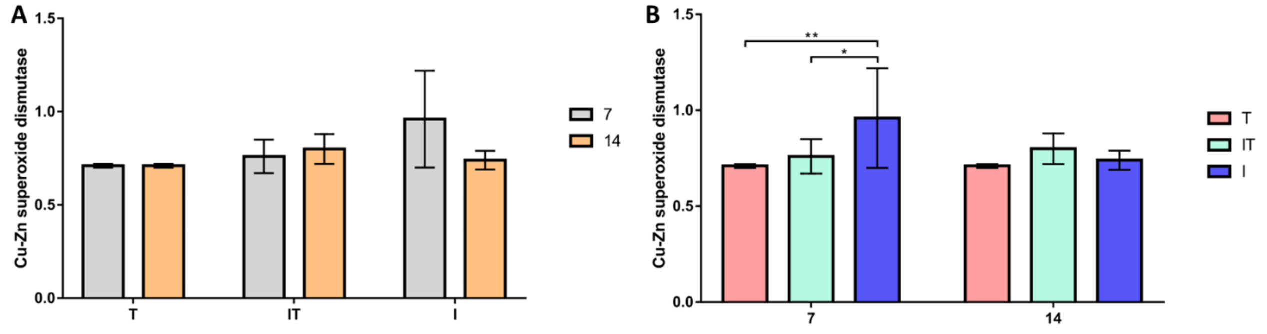

Supplementary Figure. S2. Mean values for the relative gene expression of cytoplasmatic Cu-Zn superoxide dismutase: (A) comparisons between sampling occasions (on day 7 and 14) within each group and (B) comparisons between groups at each sampling occasion. *p < 0.05; **p < 0.01; Treated group (T), *N. ceranae*-infected and treated group (IT), *N. ceranae*-infected group (I) and non-infected group (NI).

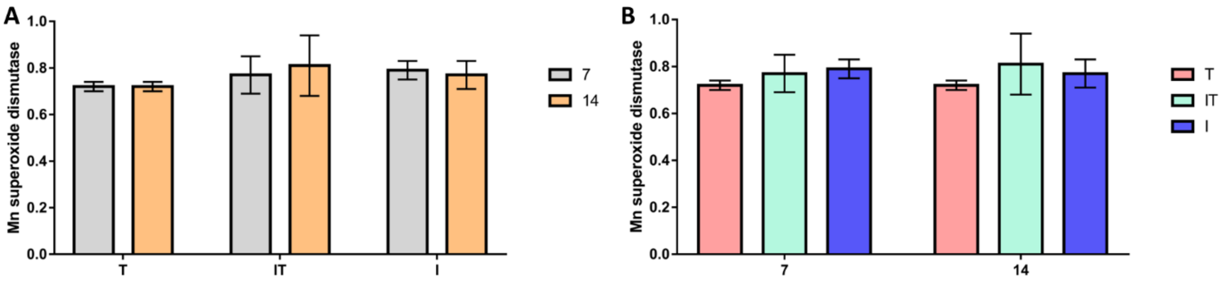

Supplementary Figure. S3. Mean values for the relative genes expression of mitochondrial Mn superoxide dismutase: (A) comparisons between sampling occasions (on day 7 and 14) within each group and (B) comparisons between groups at each sampling occasion. Treated group (T), *N. ceranae*-infected and treated group (IT), *N. ceranae*-infected group (I) and non-infected group (NI).

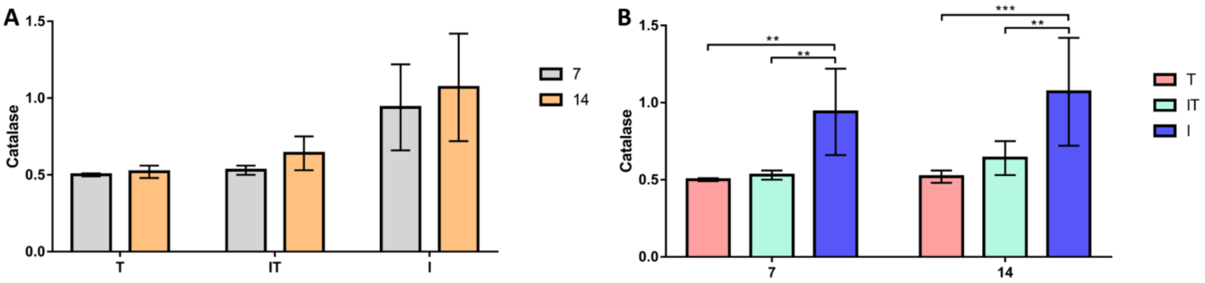

Supplementary Figure. S4. Mean values for the relative gene expression of catalase: (A) comparisons between sampling occasions (on day 7 and 14) within each group and (B) comparisons between groups at each sampling occasion. **$p < 0.01$; ***$p < 0.001$; Treated group (T), *N. ceranae*-infected and treated group (IT), *N. ceranae*-infected group (I) and non-infected group (NI).

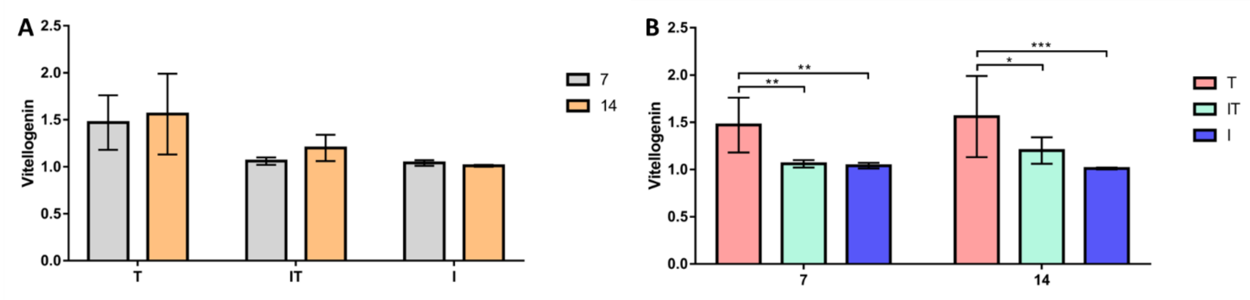

Supplementary Figure. S5. Mean values for the relative gene expression of vitellogenin: (A) comparisons between sampling occasions (on day 7 and 14) within each group and (B) comparisons between groups at each sampling occasion. *$p < 0.05$; **$p < 0.01$; ***$p < 0.001$; Treated group (T), *N. ceranae*-infected and treated group (IT), *N. ceranae*-infected group (I) and non-infected group (NI).
